# Selected Properties of Cement Bound Spruce and Larch Bark Bio-Aggregates

**DOI:** 10.3390/polym13244438

**Published:** 2021-12-17

**Authors:** Johannes Urstöger, Marius Cătălin Barbu, Thomas Pacher, Alexander Petutschnigg, Johannes Jorda, Eugenia Mariana Tudor

**Affiliations:** 1 Forest Products Technology and Timber Construction Department, Salzburg University of Applied Sciences, Markt 136 a, 5431 Kuchl, Austria; jurstoeger.htw-m2021@fh-salzburg.ac.at (J.U.); cmbarbu@unitbv.ro (M.C.B.); tpacher.htw-m2021@fh-salzburg.ac.at (T.P.); alexander.petutschnigg@fh-salzburg.ac.at (A.P.); johannes.jorda@fh-salzburg.ac.at (J.J.); 2 Faculty of Furniture Design and Wood Engineering, Transylvania University of Brasov, B-dul. Eroilor nr. 29, 500036 Brasov, Romania; 3 Institute of Wood Technology and Renewable Materials, University of Natural Resources and Life Sciences (BOKU), Konrad Lorenz-Straße 24, 3340 Tulln, Austria

**Keywords:** tree bark, spruce, larch, cement-bonded bark-based composite materials

## Abstract

The aim of this study is to investigate the suitability of spruce and larch bark for the production of cement-bonded composites. At the beginning of this research, the curing behaviour of the admixtures was quantified with temperature profiles when testing spruce, larch, pine and poplar bark, to determine the compatibility between the components of the bio-aggregates, to analyse the cement curing and to establish which bark species should be successfully included in cement bonded composites. Considering the results, it was observed that the average densities of 600–700 kg/m^3^ of bio-aggregates are 40–55% lower than that of established products on the market, although spruce and larch bark are in a similar range. The situation is different for the compressive strength, as larch bark showed up to 30% higher values than spruce bark. This study revealed also different hardening characteristics of the two cement types used as binders for spruce and larch bark. The results of this study demonstrated that tree bark of *Picea abies* and *Larix decidua* Mill. can be successfully utilized for the production of a cement-bonded composite material.

## 1. Introduction

Using lignocellulosic resources combined with cement as raw materials for construction is an interesting topic of research. Wood-cement composites (WCC) or wood chip concrete consist of cement, water and wood chips. WCC are used for the production of water- and fire-retardant as well as sound-absorbing materials for exterior construction applications. These are included in the group of wood-cement panels (WCP) made from wood chips or particles of ligno-cellulosic residues or fibres [1]. Cement-bonded wood-based materials have been manufactured industrially for decades and include products such as particleboard, fibreboard, wood wool panels, as well as sound insulation elements and hollow blocks for primary use in construction [1]. WCC are characterized by high weather resistance, protection against insect and fungal attack, fire resistance and sound-absorbing properties [2,3,4]. These composites have no formaldehyde emissions due to the inorganic binder and can be manufactured with a certain amount of recycled material [5]. Due to its exothermic reaction behaviour, the setting of cement can be monitored by measuring the hydration temperature. The curves consist normally of three phases: (a) initial temperature rise, (b) dormant period (temperature remains constant or slight decreases) and (c) cement hardening [6]. WCC have been used since the beginning of the 20th century, mostly as sawdust concrete and wood wool cement bonded panels [1,7,8,9]. Due to its low density, WCC are included in the category of lightweight concrete composites. Depending on the mixing and compression ratio, a bulk density between 400 and 1700 kg/m^3^ can be achieved [10]. The fundamental incompatibility of wood and cement makes the manufacture of WCC difficult. Influencing factors, such as the different pH value of wood and cement, the varying composition and amount of wood extracts, and the polysaccharide structure of the cellulose and hemicellulose of different wood species have a predominantly negative effect on the hydration phases of cement [11]. Due to the chemical structure of cement and cellulose, hydrogen bonding and/or hydroxyl bridges influence the mix of these components [12]. Hemicelluloses, starches, sugars, phenols and acids as main and secondary wood components tend to hinder cement hydration, determining a longer setting time and limiting the strength of the cementitious composite due to the micro-fracturing of the matrix during cement hydration [12,13]. Cement hardening is an exotherm process and a temperature time diagram can be used to assess the compatibility between lignocellulosic substances and cement [5]. Such graphs are interesting as, for example, T_max_ (maximum slope during cement setting phase), t_max_ (time to T_max_) and I-Index (inhibitory index) [14,15,16]. The physical and mechanical properties of WCC are significantly influenced by wood species, particle treatments and mix proportions [17]. In addition to wood particles, wood wool [14] and wood shavings [18], tree bark has been studied in cement composites. Bark is a by-product of the wood industry and is widely available [19] at a low cost. Bark is the external protective tissue that surrounds the vascular cambium zone of trees and possesses various functions [20]. Among them can be count protection of plant stems, aeration of stems in some species, water storage, sap transport, and mechanical support for tree stems [21]. It is estimated that the annual roundwood produced worldwide is approximately 3.6 × 10^9^ m^3^, 60% of which is used as firewood. Assuming an average bark content of 10%, around 150–180 × 10^6^ m^3^ bark is produced annually by industrial wood logs [19]. Except for energy generation, tree bark can be used as thermal insulating [22,23,24] and sound absorbing material [25,26] as fire-retardant panels [27], as single-layer [28,29] or 3-layers particleboard [30], medium density fibreboards [31] and oriented strand boards [32,33].

Cement-based bio-aggregates with tree bark were studied by [34] as environmentally friendly insulation panels. The authors stated that certain bark species, due to their large amounts of extractives, can hinder the cement setting. The effects of *Eucalyptus globulus* bark fibre in traditional concrete mechanical properties were evaluated by [35]. The results of this study promote the practical use of bark fibre of *Eucalyptus globulus* in numerous areas of concrete applications such as, roadways, residential, agricultural, commercial, tunnelling, precast concrete products, airports, warehouses, etc. Waste granular cork composites with slag cement were analysed for their thermo-mechanical and physical properties by [36] and it was stated that the composite could be used in some applications such as non-load carrying elements and insulation elements. Karade, Irle and Maher [37] studied the compatibility of cork (*Quercus suber*) granules with cement for the manufacture of lightweight cementitious composites. The study focused on the effects of extractives, particle size and density of the cork granules on the properties of cork-cement composites. At 10% cork in the admixture, only the extractives had an influence on hydration behaviour. At 20% and 30% cork, the compatibility between these materials was determined by particle size and density. Nevertheless, according to this study, cork granules were found to be compatible with cement.

Eusebio et al. [38] investigated the use of *Cryptomeria japonica* bark for cementitious composites. The results of this study emphasized the inhibitory role of the bark on the hydration of cement. With the addition of magnesium chloride and sodium silicate, the hydration behaviour of the composite improved, with a slight enhancement of compressive strength. The addition of magnesium chloride positively influenced the bending strength, stiffness and internal bond, for the admixtures using the conventional hot-pressing method. In the case of steam injection, pressing the additive sodium silicate was more adequate. However, according to this study, the properties of these bark aggregate cement composites did not meet the standard specifications.

The aim of the study is to analyse the combability between cement and four different soft-/hardwood bark species (spruce, larch, pine and poplar) on the hydration behaviour, as well as the suitability, of bark-cement aggregates as structural materials.

## 2. Materials and Methods

The raw materials used for the trials were spruce (*Picea abies*), larch (*Larix decidua* Mill.), pine (*Pinus sylvestris*) and poplar (*Populus alba*) bark, sourced from the sawmill, Rupert Deisl Co. in Adnet, Austria. The bark material was shredded with a R40 four-spindle shredder (Untha shredding technology Co., Kuchl, Austria), dried to a moisture content (MC) of 14–15% in a fresh air/exhaust air drying system (Hildebrand-Brunner Co., Hannover, Germany) and then fractionated into particles <3 mm, 3–7 mm and 7–12 mm. The binder applied was Portland-composite cement CEMIIA-LL42.5N’PROFI-CEM’ from Zementwerk Leube Co. (St. Leonhard, Austria) and in addition recycled material from cement-bonded bark with a particle size <1–>4 mm, which originated from preliminary trials. For the hydration tests, CEMI52.5R ‘Premium’ (Zementwerk Leube) was also included for the experimental trials.

### 2.1. Sample Preparation for Hydration Behaviour

**Figure 1:**
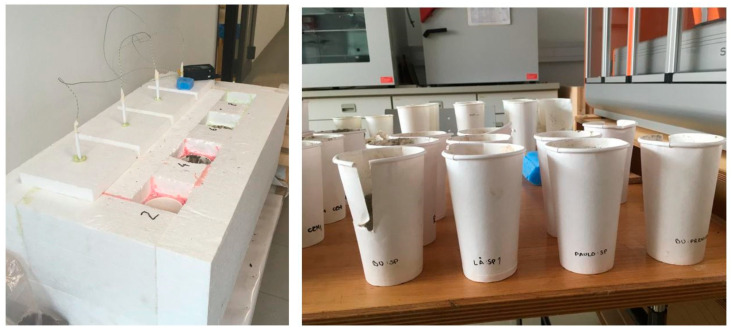
Insulation box (**left**) and paper cups with bark-cement mixture (**right**) for hydration temperature measurements.

The measurements of the hydration temperature curves are based on Wei et al. [39]. The setup for the hydration behaviour was as follows: 15 g of kiln-dried bark powder (spruce, larch, pine, poplar), 200 g of cement (CEMIIA-LL42.5N, CEMI52.5R) and 90 g of water were mixed in a paper cup (W/B value of 0.45, three samples per bark type). To avoid climatic influences, the samples were placed in a thermally insulated polystyrene test box under constant ambient climate (20 (±1) °C; 65% rel. humidity). The thermocouple connected to the data logger Testo 177 T4 (Testo GmbH, Vienna, Austria) were inserted in the middle of the sample (Figure 1) to measure the temperature over a total duration of 45–72 h. The time interval was set to 5 min. In addition, a pure cement paste sample was prepared as a reference. In order to map comparative values to the hydration temperature curves, wood chips and pure cement paste samples were additionally prepared in both tests. In the trials with CEM II, the control samples were produced with spruce chips and recycled material in accordance with industrial procedures, while with CEM I, the group spruce particles, were processed purely with spruce wood chips.

### 2.2. Sample Preparation for Determination of Density and Compressive Strength

Initially, the MC of the bark types was raised from 14–15% to approximately 100% with 24 h water storage in order not to change nor to influence the water binder value (W/B value). Subsequently, the bark particles were mixed dry with cement and recycled material in a single-shaft ploughshare mixer ENT type WHB–75 and the required proportion of water was added. Two test groups depending on the bark fractions were used (Table 1). Test group 1 (TG1) was produced with bark particle size <3–12 mm, test group 2 (TG2) with ground bark particles of <3–7 mm. Two different compression methods were tested for the bark-cement composites using screw clamps (SC) as well as a hydraulic press Höfer HLOP 280 (Höfer, Taiskirchen, Austria) (HP) (Table 1).

**Figure 2:**
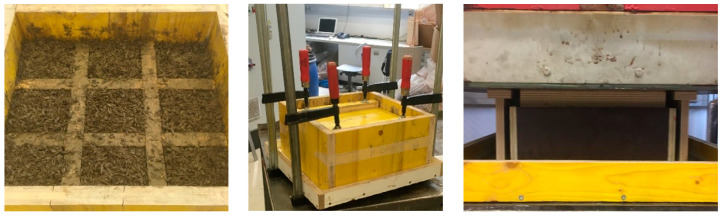
Test specimens of 100 × 100 × 100 mm^3^ in a forming frame (**left**); Compaction by screw clamps (**middle**) and compaction by hydraulic press (**right**).

Based on a target density of 750 kg/m^3^ and a size per test sample of 100 × 100 × 100 mm^3^ (Figure 2), each sample should consist of 0.75 kg of mixed material. In fact, 0.93 kg per specimen was obtained, which is due to the amount of water needed to bring the bark to 100% moisture content.

Depending on the compaction type (SC or HP), the cement bound bio-aggregates were closed in moulds for 48 h with SC (9 individual test samples) or cold pressed (at 20 °C) for 10 min with the Höfer HLOP 280 hydraulic laboratory press at 12–15 bar (one block of 9 test specimens as in Figure 2 left). For curing, all samples were stored in a standard climate at 20 °C and 65% relative air humidity for 28 days. The finished mixture had a W/B value of 0.5. The densities of the test specimens were measured according to EN 323:2005 [40]. The compressive strength was determined according to EN826:2013 [41].

**Figure 3:**
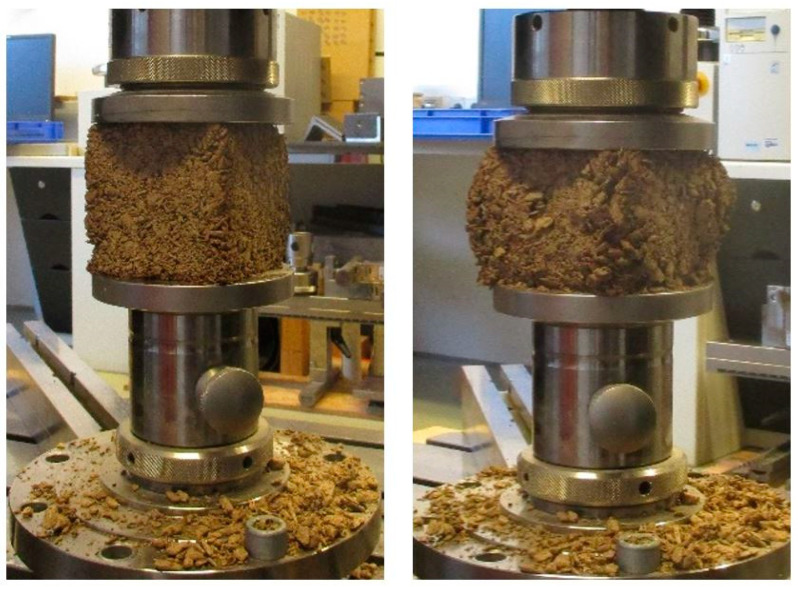
Compressive strength measurement at 10% compression of the 100 × 100 × 100 mm^3^ specimen using universal testing machine Zwick/Roell 250.

The compressive strength (σ_m_) or the compressive tension at 10% compression (σ_10_) were measured with universal testing machine Zwick/Roell 250 (Ulm, Germany) (Figure 3).

## 3. Results and Discussion

First the hydration behaviour of cement and bio-aggregates based on spruce, larch, pine and poplar bark will be analysed and discussed. Second, a quantitative analysis of the thermal behaviour of water-cement-tree bark admixtures will be presented. Third, density and compressive strength of the test groups, as decisive factors, will be analysed and discussed to prove their suitability as prospective construction materials.

### 3.1. Hydration Temperature Measurements

**Figure 4:**
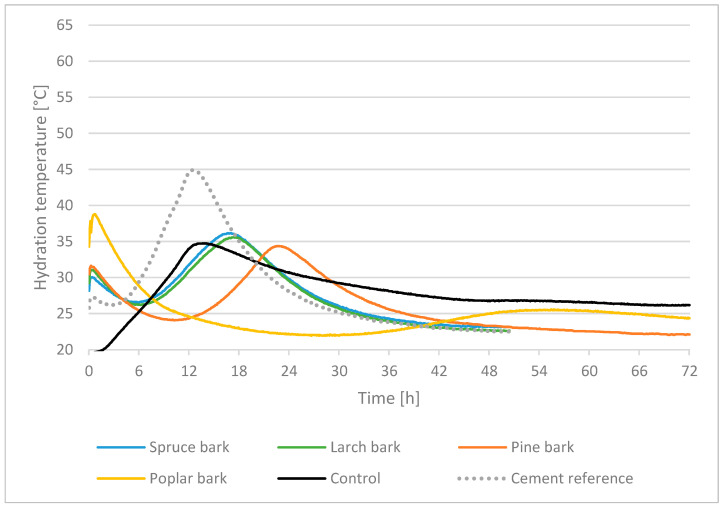
Comparison of the thermal behaviour during hydration of cement reference with admixtures of spruce, larch, pine, poplar bark and control with CEM II A–LL 42.5 N.

**Figure 5:**
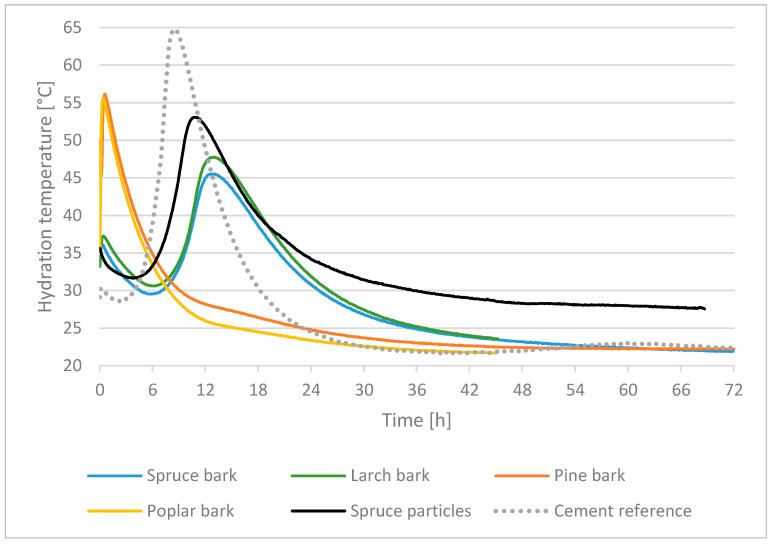
Comparison of the thermal behaviour during hydration of cement reference with admixtures of spruce, larch, pine, poplar bark and spruce particles with CEM I 52.5 R.

According to Wei et al. [39], a first temperature peak was measured within the first hour of hydration reaction for all four bark species and two cement types (CEMIIA–LL42.5N, CEMI52.5R) in both tests (CEM I and CEM II). There is a significant difference between softwood and hardwood bark regarding hydration reaction of the admixture, excepting pine bark in the trial with CEM I. All softwood bark measurements showed a second temperature peak in the interval of 16–23 h. This is consistent with the findings of Wei et al. [39]. It suggests that the differences in chemical composition between coniferous and hardwood bark may be responsible. The measurement results are shown in Table 2 and Figure 4 and Figure 5. In order to compare the results, the “Inhibitory Index” (I-Index) (Formula 1) based on Hofstrand et al. [16] was applied. By means of this I-Index it is possible to compare mixtures with different lignocellulosic materials for the use of cement composites

(1) $$I=\left[\left(\frac{t_{2}-{\;t\prime }_{2}}{{t\prime }_{2}}\right)\;\left(\frac{{T\prime }_{2}-{\;T}_{2}}{{T\prime }_{2}}\right)\;\left(\frac{S\prime -\;S}{S}\right)\right]$$

where:

I inhibitory index (I-Index)
$t_{2}$ time required for the wood–cement mixture to reach maximum temperature [h]
${t\prime }_{2}$ time required for cement to reach maximum temperature [h]
$T_{2}$ maximum temperature of the wood-cement mixture [°C]
${T\prime }_{2}$ maximum temperature of cement [°C]
S slope of the temperature curve of the wood-cement mixture>
S’ slope of the temperature curve of cement

Considering the individual hydration curves in tests with Portland Cement CEM II (Figure 4), the assumption is that spruce, larch and pine bark initially had a slight retarding effect on hydration, since the temperatures dropped after the first peak before they rose to T_max_. Furthermore, T_max_ was reached significantly behind the temperature achieved by cement reference. In the case of poplar bark, an inhibiting or preventing effect was shown with reference to [15] due to the sharply falling temperature curve. In contrast to spruce, larch and pine bark and the research of [34], only one temperature peak was reached at the beginning of the hydration reaction for the samples manufactured with spruce particles. A comparison of the three bark types of coniferous species with the control samples (Figure 4) shows that they, especially the hydration temperatures for spruce and larch bark, are close to the values of the industrial control samples with regard to T_max_ and t_max_. This resulted in a difference in temperature and time between the hydration of spruce bark and control of 1.5 °C at 3.1 h and larch bark and control of 0.9 °C at 3.8 h. Therefore, only a small deviation of 0.9–1.5 °C is present, but regarding the first peak, there was a large difference at the beginning of hydration reaction.

Based on the apparent inhibitory effect of polysaccharides in bark on the hydration reaction displayed by the temperature plot, the inhibitory index (I-Index) [16] was as follows: for spruce bark 3.78, for larch bark 4.88, for pine bark 8.74 and for control 1.05 (Table 2). As described by [37], low values of I-Index for conifers have a better expected tolerance, whereby especially spruce and larch bark appear to be more suitable in admixtures with cement, although the best index could be achieved with the data of the control.

Analysing the individual hydration curves in tests with cement CEM I type, it can be observed that the hydration temperature curves for spruce and larch bark are almost similar to those obtained with CEM II, whereby T_max_ increased by approximately 10–20 °C due to the different cement types. T_max_ was reached 3–4 h min earlier for spruce and larch bark, which can be attributed to a higher clinker content [42] of the cement (Figure 4). The hydration temperature curve for pine was completely different in comparison to the other coniferous barks. While the hydration of pine bark was similar to the other softwood bark types in tests with CEM II, in admixture with CEM I it is almost identical to the data of poplar bark. The reasons for this are difficult to determine, although it is suspected that fluctuations in the ingredient amounts could be decisive, whereby cement hydration is inhibited or prevented. To compare spruce bark with spruce wood particles, an additional measurement was performed with spruce wood particles. With T_max_ of 53.1 °C at t_max_ of 10.7 h (Figure 5), the hydration with spruce particles is similar to the cement reference, but T_max_ differs by 11.7°C. In comparison with spruce bark (T_max_ of 45.5 °C, t_max_ at 10.8 h), spruce particles yielded better values (temperature difference of 7.6 °C, time difference of 2.1 h). It should be considered that T_max_ may not reflect with precision any hindrance of cement curing. This is due to the mass of cement reference paste being less than the lignocellulosic aggregate paste [5]. The heat in a testing sample is sourced from cement, while its weight is constant. Therefore, the T_max_ of the samples with tree bark will be lower than that of cement reference, due to the fact that the bio-aggregate will absorb a part of the heat generated by the hydration reactions. Moreover, an increase in T_max_ is justified by the deceleration of cement hydration in lignocellulosic admixture.

The following I-Index is determined for CEM I: for spruce bark 5.70, for larch bark 4.18 and for spruce particles 2.23. Based on the characteristics described by [43], spruce and larch bark seem to be suitable in cement bound bio-aggregates, whereby the lowest index could be achieved with spruce particles.

### 3.2. Densities and Compressive Strengths

**Figure 6:**
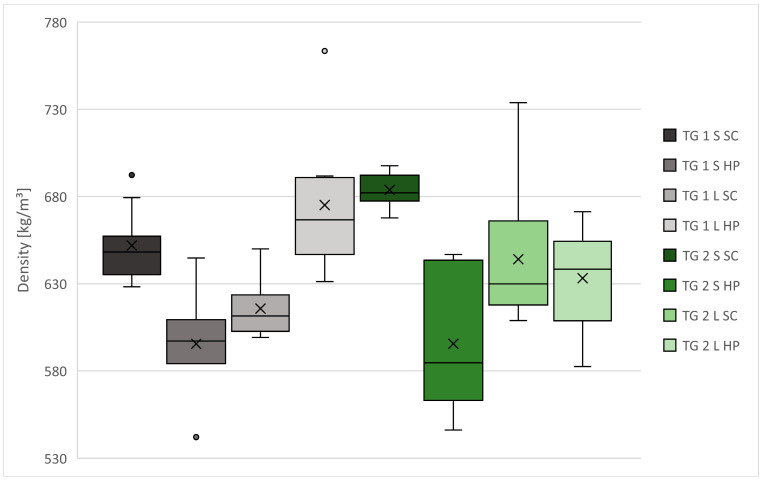
Density of 100 × 100 × 100 mm^3^ specimens. Note: TG, test group; S, spruce bark; L, larch bark; SC, screw clamps; HP, hydraulic press.

**Figure 7:**
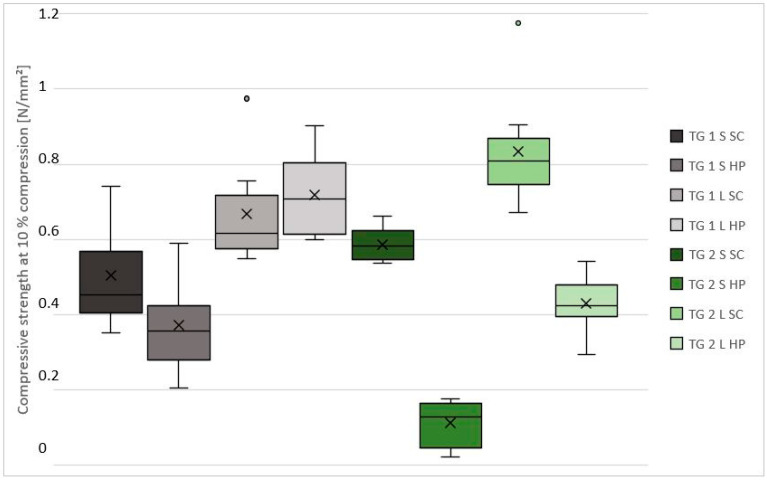
Compressive strengths at 10% compression of the 100 × 100 × 100 mm^3^ specimens. Note: TG, test group; S, spruce; L, larch; SC, screw clamps; HP, hydraulic press.

The results of the average density and compressive strength measurements are shown in Table 3, Figure 6 and Figure 7.

The average densities of the manufactured test specimens vary between 595–684 kg/m^3^ for spruce bark and 616–675 kg/m^3^ for larch bark (Table 3, Figure 6). Statements about the influencing parameters are difficult to establish. As shown in Figure 6, higher mean densities of 652 (TG 1) and 684 kg/m^3^ (TG2) were achieved for spruce bark in both test groups by compaction with SC, while the maximum mean density of larch bark in TG1 (675 kg/m^3^) was obtained by compaction with HP and a similar level of about 640 kg/m^3^ in TG2. This trend was also observed for the different bark fractions, as spruce and larch bark produced higher values depending on the test group and compaction. The highest and most constant values could be achieved in TG 2SSC (density of 668–698 kg/m^3^).

Compared to the target density, nearly all values are lower, which leads to the assumption that the evaporation of water during curing could be the reason. Although the amount of water needed for cement hydration was taken into the mixing ratio, the amount needed to bring bark from 14–15% to approximately 100% moisture was not considered. As a result, more water could have been used for evaporation.

The average compressive strengths at 10% compression of the manufactured test specimens vary between 0.11–0.59 N/mm^2^ for spruce and 0.43–0.83 N/mm^2^ for larch bark (Table 3, Figure 7). As was already the case with the density, influencing parameters are difficult to fix, whereby larch bark showed up to 30% higher compressive strength than spruce bark, irrespective of fraction and compaction. With one exception, higher values for the compressive strength can be obtained by compacting with SC. Differences between the individual bark fractions are particularly visible in TG1LHP and TG2LHP, as the assumed improvements in compressive strength due to the fraction change from <3–12 mm to <3–7 mm could not be conclusively confirmed. The most constant results were obtained in TG2SSC with a compressive strength of 0.54–0.66 N/mm^2^, as was already the case with the density determination.

Considering the correlation of the compressive strength at 10% compression to the density of the individual TG, both groups show positive correlations. This is in accordance to the general fact [31], that density has a considerable influence on the mechanical properties of composite materials.

**Figure 8:**
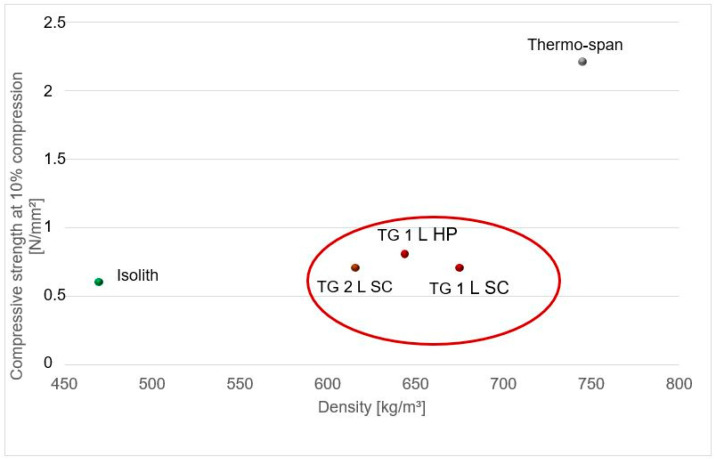
Comparison of compressive strengths at 10% compression between industrial manufactured cement bonded wood composites Isolith and Thermo-span with larch-bark-cement admixtures.

The cement-bonded bark composites are included in the category of lightweight mineral compounds. From all bark species chosen for this study, the compressive strength of larch bark bio-aggregates with cement was from 1 to 8 times higher compared to the composites manufactured with spruce bark. A comparison of the compressive strength of the cement-bark composites with industrial manufactured lightweight products Isolith [44] and Thermospan [45] is depicted in Figure 8.

Because the bark-aggregate concrete had the highest values of compression strength at 10% compression, three samples of larch-bark-cement admixtures (TG1LHP, TG1LSC and 3–7 mm, TG2LSC) were chosen for this comparison. The compressive strength of this group, at densities between 616 and 675 kg/m^3^, was 0.7 to 0.8 N/mm^2^ (standard deviation 0.1 N/mm^2^), 15 % higher compared to Isolith material (cement bonded wood wool panel, 0.6 N/mm^2^, standard deviation 0.06 N/mm^2^) [44] and about 70% lower than that of thermo-span product (cement bonded wood chips composite, 2.2 N/mm^2^, standard deviation 0.5 N/mm^2^) [45].

## 4. Conclusions

Hydration behaviour and compressive strength of the cement bound bio-aggregates showed that spruce and larch bark can be considered suitable for the production of lightweight cement-bonded composites. Larch bark, in particular, has the greatest potential by compaction using screw clamps. This is confirmed not only by the results of density, but by the compressive strength and the hydration measurements. Although the compressive strength of spruce bark is lower than that of larch, the most constant values were achieved for the latter, as well as for the density; the high availability would also speak in favour of this bark type. The assumed improvements in compressive strength due to a fraction change from <3–12 mm (TG1) to <3–7 mm (TG2) could not be conclusively confirmed, as spruce and larch bark yielded higher values depending on the test group and compaction. Compaction by hydraulic press resulted with one exception in the lowest values. Further research should focus on the production process parameters of cement bonded bark composites, including the use of additives, such as aluminium sulphate, magnesium chloride or sodium silicate.

In order to gain further knowledge regarding the compatibility of bark and cement, an extended series of hydration temperature measurements would be recommended to investigate different types of bark and cement to perform a certain pre-selection. Additionally, chemical constituent analysis is mandatory to evaluate the effect of the varying range of bark constituents on the cement bark combability.

Furthermore, pre-treatment of bark with additives in dosages up to 5 % such as calcium chloride (CaCl_2_) or sodium hydroxide (NaOH) might open new possibilities, as the low pH value of tree bark could be better adapted to the high value of cement.

The present study reveals the potential of the two central European native conifers larch and spruce bark types and its suitability for the production of cement-bonded composites.

## Figures and Tables

**Table 1:** Experimental design for bark-based cement-bonded composites. Note: SC, screw clamps; HP, hydraulic press.

	Bark	Test Samples	Compaction	Cement (%)	Water (%)	Particle Size (%)			Recycling Material (%)
						<3 mm	3–7 mm	7–12 mm	<1–>4 mm
TG 1 <3–12 mm	spruce	n = 17	SC	39.26	19.64	9.86	12.30	12.30	6.64
		n = 5	HP						
	larch	n = 9	SC						
		n = 9	HP						
TG 2 <3–7 mm	spruce	n = 9	SC	39.26	19.64	9.86	24.60	-	6.64
		n = 6	HP						
	larch	n = 9	SC						
		n = 9	HP						

**Table 2:** Hydration temperature of tree bark compared to control sample, aggregate with spruce particles and cement reference. Note: T_max_, maximum temperature; t_max_, time required to achieve T_max_; I-Index, inhibitory index.

		Spruce Bark	Larch Bark	Pine Bark	Poplar Bark	Control	Spruce Particles	Cement Reference
CEM II	T_max_ (°C)	36.2	35.6	34.4	38.8	34.7	-	44.9
	t_max_ (h)	16.7	17.4	22.5	0.7	13.6	-	12.3
	I-Index	3.78	4.88	8.74	-	1.05	-	-
CEM I	T_max_ (°C)	45.5	47.8	56.1	55.6	-	53.1	64.8
	t_max_ (h)	12.8	13.0	0.6	0.5	-	10.7	8.4
	I-Index	5.70	4.18	-	-	-	2.23	-

**Table 3:** Density and compressive strength of cement reference with admixtures of spruce, larch, with two types of bark particle sizes. Note: SC, screw clamps; HP, hydraulic press; standard deviation in parentheses.

	Bark	Compaction	Test Samples	Density (kg/m^3^)	Compressive Strength (N/mm^2^)
Test group 1 <3–12 mm	spruce	SC	n = 17	652 (21)	0.504 (0.133)
		HP	n = 5	595 (37)	0.371 (0.148)
	larch	SC	n = 9	616 (16)	0.669 (0.137)
		HP	n = 9	675 (39)	0.719 (0.108)
Test group 2 <3–7 mm	spruce	SC	n = 9	684 (9)	0.586 (0.043)
		HP	n = 6	596 (41)	0.111 (0.062)
	larch	SC	n = 9	644 (41)	0.834 (0.145)
		HP	n = 9	633 (29)	0.430 (0.070)

## Data Availability

Not Applicable.

## References

[1] Paulitsch M., Barbu M.C. (2015). Holzwerkstoffe der Moderne, 1. Auflage.

[2] Wassilieff C. (1996). Sound absorption of wood-based materials. Appl. Acoust..

[3] Dunky M., Niemz P. (2002). Holzwerkstoffe und Leime: Technologie und Einflussfaktoren.

[4] Botterman B., La Doudart de Grée G., Hornikx M., Yu Q.L., Brouwers H. (2018). Modelling and optimization of the sound absorption of wood-wool cement boards. Appl. Acoust..

[5] Pereira C., Caldeira J.F., Irle M., Ferreira J.M. (2006). Characterizing the setting of cement when mixed with cork, blue gum, or maritime pine, grown in Portugal I: Temperature profiles and compatibility indices. J. Wood Sci..

[6] Moslemi A.A., Lim Y.T. (1984). Compatibility of southern hardwoods with Portland cement. For. Prod. J..

[7] Patel M., Patel K., Patel A., Prajapati R., Koshti U. Study of Sawdust Concrete Properties as Construction Materials. Proceedings of the 3rd International Conference on Multidisciplinary Research & Practice.

[8] Acharya S., Neupane U., Adhikari S. Strength Optimization of Sawdust Concrete through Cement Variation. Proceedings of 8th IOE Graduate Conference.

[9] Plotnikov N., Kochetkov I. (2021). Possibility of using sawdust in sawdust concrete. E3S Web Conf..

[10] Sonntag F. (2003). Wandbauten mit Schalungssteinen aus Holzspanbeton.

[11] Vaickelionis G., Vaickelioniene R. (2006). Cement hydration in the presence of wood extractives and pozzolan mineral additives. Ceram. Silik..

[12] Frybort S., Mauritz R., Teischinger A., Müller U. (2008). Cement bonded composites—A mechanical review. BioResources.

[13] Lin X., Silsbee M.R., Roy D.M., Kessler K., Blankenhorn P.R. (1994). Approaches to improve the properties of wood fiber reinforced cementitious composites. Cem. Concr. Res..

[14] Weatherwax R.C., Tarkow H. (1964). Effect of Wood on Setting of Portland Cement. For. Prod. J..

[15] Sandermann W., Brendel M. (1956). Studien über mineralgebundene Holzwerkstoffe—Zweite Mitteilung: Die „zementvergiftende” Wirkung von Holzinhaltsstoffen und ihre Abhängigkeit von der chemischen Konstitution. Holz als Roh-und Werkstoff.

[16] Hofstrand A.D., Moslemi A., Garcia J.F. (1984). Curing characteristics of wood particles from nine northern Rocky Mountain species mixed with Portland cement. For. Prod. J..

[17] Garcez M.R., Garcez E.O., Machado A.O., Gatto D.A. (2016). Cement-Wood Composites: Effects of Wood Species, Particle Treatments and Mix Proportion. Int. J. Compos. Mater..

[18] Li M., Khelifa M., El Ganaoui M. (2017). Mechanical characterization of concrete containing wood shavings as aggregates. Int. J. Sustain. Built Environ..

[19] Tudor E.M., Zwickl C., Eichinger C., Petutschnigg A., Barbu M.C. (2020). Performance of softwood bark comminution technologies for determination of targeted particle size in further upcycling applications. J. Cleaner Prod..

[20] Giannotas G., Kamperidou V., Barboutis I. (2021). Tree bark utilization in insulating bio-aggregates: A review. Biofuels Bioprod. Biorefin..

[21] Pásztory Z., Mohácsiné I.R., Gorbacheva G., Börcsök Z. (2016). The utilization of tree bark. BioResources.

[22] Kain G., Tudor E.M., Blanchet P. (2020). Bark Thermal Insulation Panels: An Explorative Study on the Effects of Bark Species. Polymers.

[23] Gößwald J., Barbu M.C., Petutschnigg A., Tudor E.M. (2021). Binderless Thermal Insulation Panels Made of Spruce Bark Fibres. Polymers.

[24] Kristak L., Ruziak I., Tudor E.M., Barbu M.C., Kain G., Reh R. (2021). Thermophysical Properties of Larch Bark Composite Panels. Polymers.

[25] Tudor E.M., Dettendorfer A., Kain G., Barbu M.C., Réh R., Krišťák L. (2020). Sound-Absorption Coefficient of Bark-Based Insulation Panels. Polymers.

[26] Tudor E.M., Kristak L., Barbu M.C., Gergeľ T., Němec M., Kain G., Réh R. (2021). Acoustic Properties of Larch Bark Panels. Forests.

[27] Tudor E.M., Scheriau C., Barbu M.C., Réh R., Krišťák L., Schnabel T. (2020). Enhanced Resistance to Fire of the Bark-Based Panels Bonded with Clay. Appl. Sci..

[28] Muszinsky Z., McNatt J. (1984). Investigations on the use of spruce bark in the manufacture of particleboard. For. Prod. J..

[29] Calve L., Shields J., Gravel M. (1986). Maximizing aspen poplar residues utilization for waferboard production. For. Prod. J..

[30] Claude M., Yemele N., Blanchet P., Cloutier A., Koubaa A. (2008). Effects of bark content and particle geometry on the physical and mechanical properties of particleboard made from black spruce and trembling aspen bark. For. Prod. J..

[31] Xing C., Deng J., Zhang S.Y. (2007). Effect of thermo-mechanical refining on properties of MDF made from black spruce bark. Wood Sci. Technol..

[32] Nishimura T. (2015). Chipboard, oriented strand board (OSB) and structural composite lumber. Wood Composites.

[33] Igaz R., Krišťák L., Ružiak I., Gajtanska M., Kučerka M. (2017). Thermophysical properties of OSB boards versus equilibrium moisture content. BioResources.

[34] Karade S.R. (2015). Potential of Cork Cement Composite as a Thermal Insulation Material. Key Eng. Mater..

[35] Mansilla C., Pradena M., Fuentealba C., César A. (2020). Evaluation of Mechanical Properties of Concrete Reinforced with Eucalyptus globulus Bark Fibres. Sustainability.

[36] Merabti S., Kenai S., Belarbi R., Khatib J. (2021). Thermo-mechanical and physical properties of waste granular cork composite with slag cement. Constr. Build. Mater..

[37] Karade S.R., Irle M., Maher K. (2006). Influence of granule properties and concentration on cork-cement compatibility. Holz als Roh-und Werkstoff.

[38] Eusebio D.A., Yamauchi H., Sasaki H., Kawai S. Bark cement composites. Proceedings of the Third Pacific Rim Bio Based Composites Symposium.

[39] Wei Y.M., Guang Z.Y., Tomita B. (2000). Hydration behavior of wood cement-based composite I: Evaluation of wood species effects on compatibility and strength with ordinary Portland cement. J. Wood Sci..

[40] EN 323:1993 (1993). Wood-Based Panels—Determination of Density.

[41] EN 826:2013 (2013). Thermal Insulating Products for Building Applications—Determination of Compression Behaviour.

[42] Dhir R.K., Ghataora G.S., Lynn C.J. (2017). Concrete-Related Applications. Sustainable Construction Materials.

[43] Stokke D.D., Wu Q., Han G. *Introduction to Wood and Natural Fiber Composites*; Wiley: Hoboken, NJ, USA, 2014. ISBN 978047071.

[44] Isolith (2021). Wood-Wool Cement Bonded Boards. http://www.isolith.com/.

[45] Thermo-Span (2021). Thermo-Span Baustoffwerk. http://thermo-span.com/.

